# Occurrence of *Serratia marcescens* in bovine intramammary infections: identification and antimicrobial susceptibility in a dairy herd

**DOI:** 10.2478/jvetres-2026-0049

**Published:** 2026-08-27

**Authors:** Małgorzata Targońska-Karasek, Wojciech Ospałek, Henryk Krukowski

**Affiliations:** Department of Animal Hygiene and Environmental Hazards, Faculty of Animal Sciences and Bioeconomy, University of Life Sciences in Lublin, 20-950 Lublin, Poland

**Keywords:** antimicrobial susceptibility, dairy herd, mastitis, milking hygiene, *Serratia marcescens*

## Abstract

**Introduction:**

Mastitis is one of the most economically significant diseases in dairy cattle, and its causation by atypical environmental pathogens is gaining attention. The study investigated the involvement of *Serratia marcescens* in intramammary infections occurring in a dairy herd and evaluated the bacterium’s antimicrobial resistance profile.

**Material and Methods:**

Between February 2024 and June 2025, 481 quarter-milk samples collected from cows on a dairy farm in eastern Poland were subjected to routine bacteriological examination. Isolates were identified by biochemical, proteomic and molecular methods and tested for antimicrobial susceptibility by automated biochemical analysis.

**Results:**

*Serratia marcescens* was isolated from the milk samples of 10 cows during different sampling periods. All isolates were consistently identified as *S. marcescens*, confirming the reliability of the applied comprehensive approach. Molecular analyses demonstrated high sequence similarity with reference strains. Antimicrobial susceptibility testing revealed uniform resistance to amoxicillin/clavulanic acid, cefuroxime, colistin and tigecycline.

**Conclusion:**

*Serratia marcescens* should be considered a relevant opportunistic pathogen in dairies, capable of causing sporadic or epidemic mastitis episodes. The findings are consistent with previous reports linking *S. marcescens* mastitis to environmental reservoirs and milking equipment contamination. Comprehensive diagnostic strategies, strict hygiene practices and targeted herd management measures are important to prevent and control mastitis caused by uncommon environmental pathogens.

## Introduction

Mastitis is one of the most common and costly diseases of dairy cattle worldwide, leading to reduced milk yield, reduced milk quality and increased treatment costs and culling costs (1, 10). Although the greatest importance is attributed to common udder pathogens such as *Staphylococcus aureus, Streptococcus uberis, Streptococcus agalactiae* and *Escherichia coli*, in recent decades, the involvement of less typical environmental microorganisms in the aetiology of mastitis has been observed, and is now increasing (1, 24).

One of the opportunistic pathogens is *Serratia marcescens* – a Gram-negative, oxidase-negative, facultatively anaerobic rod belonging to the Enterobacteriaceae family, widely distributed in water, soil, on plants and in the humid environment of dairy farms (9, 13). A special feature of this microorganism is the ability of some strains to produce the red pigment prodigiosin, but many strains isolated from animal infections do not show this pigmentation (14). Serratia marcescens has a number of virulence factors that allow it to survive in the farm environment and in the host organism. These include the ability to form biofilm on the surfaces of milking equipment and in water systems, which increases resistance to disinfectants (13, 21), as well as the production of proteolytic enzymes and haemolysins that facilitate colonisation while causing mammary gland tissue damage (16).

Both sporadic cases of mastitis caused by *S. marcescens* and epidemic outbreaks have been described in the literature as often associated with improper cleaning or disinfection of milking equipment, the use of contaminated water for rinsing milking machines or immersion of teat cups in solutions containing this microorganism (8, 13). Unlike classical mastitis pathogens, *S. marcescens* infections may be difficult to eliminate because they are naturally highly resistant to many β-lactam antibiotics and able to survive in biofilms and in moist areas of the milking environment (17, 18). The aim of this study was to identify *Serratia marcescens* as a factor responsible for cases of epidemic mastitis in a herd of dairy cows, and to attempt to indicate potential sources of infection and discuss the importance of biosecurity and equipment hygiene in preventing this type of infection.

## Material and Methods

The study was carried out between February 2024 and June 2025 on a commercial dairy farm with a herd size of 806 cows located in Lubelskie voivodeship, eastern Poland. During this period, 481 quarter-milk samples which were positive in a California mastitis test (CMT) were collected following aseptic procedures as described by the US American National Mastitis Council (11). Because some cows were sampled repeatedly, the exact number of individual animals could not be unambiguously determined based on the available dataset. Milk samples were collected from quarters that scored 1, 2 or 3 in the CMT. The milk samples used in further analyses were provided by the farm veterinarian, who was employed by the dairy operation.

The samples were cooled and transported to the laboratory, where they were subsequently cultured on 5% sheep blood agar, MacConkey agar, Sabouraud agar and Edwards–Chodkowski agar and incubated at 37°C for up to 72 h, with observations made every 24 h. Colonies displaying pure growth were Gram stained, and their cell morphology and haemolysis patterns were evaluated. Bacterial and yeast isolates were identified using standard microbiological and biochemical methods. Streptococci were identified based on colony morphology, Gram-staining characteristics, growth on Edwards agar, catalase and esculin reactions, the CAMP test, and the API 20 Strep identification system (bioMérieux). *Staphylococcus aureus* was identified according to colony morphology, hemolytic pattern, Gram-staining characteristics, catalase activity, the tube coagulase test, and the API 20 Staph identification system (bioMérieux). Gram-negative bacteria were identified using colony morphology, Gram-staining characteristics, oxidase activity, growth on MacConkey agar, and the API 20E identification system (bioMérieux). Yeasts were identified based on growth on Sabouraud dextrose agar, microscopic examination, Gram staining, and the API 20 C AUX identification system (bioMérieux). In cases of ambiguous or inconclusive identification, isolates were further analysed using the VITEK® automated identification system (bioMérieux).

*Serratia marcescens* was isolated from 10 milk samples obtained from 10 different cows. Pure, homogeneous growth of this Gram-negative bacterium was observed exclusively on 5% sheep blood agar and MacConkey agar. These strains underwent advanced identification, which included biochemical characterisation using the VITEK 2 Compact system (bioMérieux, Marcy-l’Étoile, France), MALDI-TOF mass spectrometry and PCR assays specific for *S. marcescens*.

### Identification using VITEK 2

Biochemical identification of all 10 strains was conducted employing the GN (Gram-negative) card. Bacterial suspensions were prepared by emulsifying colonies in sterile saline to achieve a turbidity equivalent to 0.5 McFarland, verified with the VITEK 2 DensiChek system (bioMérieux). Cards were filled and loaded into the analyser within 30 min of inoculum preparation, and the analyses proceeded according to the manufacturer’s instructions.

### Antimicrobial susceptibility testing (AST)

The same system was used for AST, with bacterial suspensions adjusted to 1.5×10^7^ colony-forming units/mL in 0.45% saline. The AST-N332 card was used, which contains a panel of antibiotics tailored to the evaluation of aerobic Gram-negative bacilli. The panel comprised amikacin, amoxicillin/clavulanic acid, cefepime, cefotaxime, ceftazidime, ciprofloxacin, colistin, gentamicin, imipenem, meropenem, piperacillin/tazobactam, tigecycline, tobramycin and trimethoprim/sulfamethoxazole, and the card also assessed extended-spectrum β-lactamase production. Interpretation followed European Committee on Antimicrobial Susceptibility Testing (EUCAST) guidelines, using breakpoints established for *S. marcescens* (7). Results were automatically analysed and interpreted by the system’s software.

### Identification by MALDI-TOF

For proteomic confirmation, isolates were examined using a Bruker MALDI Biotyper microflex LT/SH system (Bruker Daltonics, Bremen, Germany), paired with an *in vitro* diagnostics (IVD) library encompassing 4,194 species (version 12; Bruker Daltonics). Spectra were analysed using IVD Compass 4.2.100 software (Bruker Daltonics). Sample preparation followed the standard protocol provided by the manufacturer, employing α-cyano-4-hydroxycinnamic acid as the matrix.

### DNA procedures

Prior to DNA extraction, isolates were sub-cultured on tryptic soy agar plates to increase biomass. Extraction of DNA was then undertaken from single colonies grown for 24 h using the Tissue and Bacterial DNA Purification Kit (EURx, Gdańsk, Poland), following the manufacturer’s protocol. The quantity and purity of the extracted DNA were checked with a NanoDrop One spectrophotometer (Thermo Scientific, Wilmington, DE, USA). Samples were diluted in molecular-grade water to 20 ng/μL for subsequent analyses.

For molecular characterisation of the bacterial isolates, a multilocus sequence analysis approach was used. This strategy involved the use of four pairs of primers: one set targeting a fragment of the 16S rRNA gene and two additional sets specific for housekeeping genes. These included *dinB* (DNA damage-inducible gene B), which encodes DNA polymerase IV and was represented in this case by two sequences (*dinB1* and *dinB2*), and *rpoB*, which codes for the β-subunit of RNA polymerase. The specific primer sequences used in this study are detailed in Table 1.

**Table 1.** Primer sequences used for molecular characterisation of bacterial isolates from dairy-cow quarter milk

Gene	Forward primer	Reverse primer	Expected product length (bp)
16S rRNA	AGGGAGCTTGCCTTGGATTC	GGATGCAGTTCCCAGGTTGA	557
*dinB1*	CGCTGCACATCCGTGAAATC	TGCGCCAAATCGTATTGCTG	382
*dinB2*	GATCGCTCAGGAGATCCGTC	CCAGCTCGGGGTAGAGTTTG	452
*rpoB*	TTGTTTCTGTTGCTGCGTCG	GATTTCCTCTGGGCCAAGCT	572

Polymerase chain reactions were carried out in a total volume of 25 μL, comprising 20 ng of genomic DNA, 0.2 μM of each primer and 2× PCR Mix Plus Red (A&A Biotechnology, Gdańsk, Poland). Amplifications were performed using a Biometra TOne thermal cycler (Analytik Jena, Jena, Germany). The thermal protocol included an initial denaturation step at 95°C for 5 min; 30 cycles of denaturation at 95°C for 30 s, annealing at 60°C for 30 s and extension at 72°C for 30 s; and a final elongation step at 72°C for 5 min. The resulting amplicons were assessed by electrophoresis on a 1% agarose gel to verify expected sizes and quality. Successfully amplified products were subsequently submitted for sequencing to an external provider (Genomed, Warsaw, Poland). The generated sequences were used to perform basic local alignment search tool (BLAST) analysis to determine the nearest phylogenetic neighbours against the NCBI GenBank database.

## Results

Microbiological analysis of quarter-milk samples revealed a diverse range of microorganisms associated with intramammary infections (Table 2). The most frequently identified isolates included streptococci, coagulase-negative staphylococci (CNS) and coagulase-positive staphylococci (CPS). Additionally, *Enterococcus* spp., Enterobacteriales other than *Serratia, Micrococcus* spp., *Bacillus* spp., *Aerococcus viridans* and yeasts (*Candida* spp.) were detected.

**Table 2.** Microorganisms identified during the individual stages of microbiological analysis of dairy-cow quarter-milk samples

Month and year	Samples (n)	Cows (n)	*Serratia* sp.	CNS	CPS	MRSA	*Streptococcus* sp.	*Enterococcus* sp.	Enterobacterales other than *Serratia*	*Micrococcus* sp.	*Bacillus* sp.	*Aerococcus viridans*	*Candida* sp.	No growth	Contamination
Feb 2024	34	34	1	3	0	0	13	2	0	0	0	0	2	8	7
Mar 2024	35	35	0	1	0	0	13	0	0	0	0	0	1	9	9
Apr 2024	56	55	1	2	0	0	4	1	0	1	0	0	0	6	1
May 2024	45	44	1	5	0	0	19	1	1	1	0	0	0	10	6
Oct 2024	54	52	1	7	0	0	3	0	0	0	1	0	0	9	3
Nov 2024	47	46	1	2	1	0	17	1	2	0	0	0	1	10	6
Feb 2025	35	24	0	1	1	0	9	1	3	2	0	0	0	8	9
Mar 2025	53	52	2	7	1	1	8	2	5	1	0	0	2	19	7
Apr 2025	44	37	2	4	0	0	9	2	10	0	1	0	1	8	4
May 2025	44	40	0	12	0	0	9	2	5	0	0	1	0	9	6
Jun 2025	34	32	1	3	0	0	8	2	5	1	0	0	0	12	2

CNS – coagulase-negative staphylococci; CPS – coagulase-positive staphylococci; MRSA – methicillin-resistant *Staphylococcus aureus*

*Serratia* spp. were isolated from milk samples collected from several cows and were identified alongside other Gram-negative bacteria. These isolates were recovered during different sampling periods. A subset of samples showed no bacterial growth. Samples classified as “contaminated” (Table 2) were those yielding mixed growth of at least three morphologically distinct colony types, preventing reliable identification of a single bacterium.

*Serratia marcesce*ns was indicated in all 10 samples by biochemical identification as well as by identification using the MALDI Biotyper. The VITEK2 biochemical method classified the identification as “excellent”, which is the highest level of confidence provided by the system. It meant that the biochemical profile of the tested isolate showed a very strong match with the database pattern for the given species, making the risk of misidentification minimal. In the MALDI Biotyper the score was 2.18, which by exceeding 1.99 was in the bracket for secure to highly probable species identification. Being identified in 10 out of 481 quarter milk samples, *S. marcescens* had a low overall detection frequency. The isolates originated from different cows sampled over an extended period, suggesting sporadic occurrence rather than clustering in a short time frame.

Antimicrobial susceptibility testing revealed a largely consistent resistance pattern among all *S. marcescens* isolates (Table 3). All strains demonstrated uniform resistance to amoxicillin/clavulanic acid, cefuroxime, colistin and tigecycline. They were also all susceptible to amikacin, ciprofloxacin, gentamycin and trimethoprim/sulfamethoxazole, indicating a conserved profile of susceptibility to these agents.

**Table 3.** Minimum inhibitory concentrations against *Serratia marcescens* isolates and antibiograms of those isolates from dairy-cow quarter-milk samples

Antimicrobial agent	Isolate 1 (Feb 2024)	Isolate 2 (Apr 2024)	Isolate 3 (May 2024)	Isolate 4 (Oct 2024)	Isolate 5 (Nov 2024)	Isolate 6 (Mar 2025)	Isolate 7 (Mar 2025)	Isolate 8 (Apr 2025)	Isolate 9 (Apr 2025)	Isolate 10 (Jun 2025)
Amikacin	≤2 S	≤2 S	8 S	8 S	8 S	8 S	8 S	8 S	4 S	4 S
Amoxicillin/clavulanic acid	16 R	8 R	16 R	16 R	16 R	16 R	16 R	16 R	16 R	16 R
Cefepime	≤0.12 S	≤0.12 S	2 I	2 I	2 I	1 S	1 S	1 S	1 S	1 S
Cefotaxime	≤0.25 S	≤0.25 S	8 R	8 R	8 R	8 R	4 R	4 R	4 R	4 R
Ceftazidime	0.25 S	0.25 S	≥64 R	≥64 R	≥64 R	≥64 R	≥64 R	16 R	32 R	16 R
Cefuroxime	32 R	16 R	≥64 R	≥64 R	≥64 R	≥64 R	≥64 R	32 R	32 R	32 R
Ciprofloxacin	≤0.25 S	≤0.25 S	≤0.25 S	≤0.25 S	≤0.25 S	≤0.25 S	≤0.25 S	≤0.25 S	≤0.25 S	≤0.25 S
Colistin	≤16 R	≤16 R	≤16 R	≤16 R	≤16 R	≤16 R	≤16 R	≤16 R	≤16 R	≤16 R
Gentamicin	≤1 S	≤1 S	2 S	2 S	2 S	2 S	2 S	≤1 S	≤1 S	≤1 S
Meropenem	≤0.25 S	≤0.25 S	4 I	4 I	4 I	4 I	4 I	8 S	4 I	4 I
Piperacillin/tazobactam	≤4 S	≤4 S	64 R	64 R	≤4 S	≤4 S	≤4 S	≤4 S	≤4 S	≤4 S
Tigecycline	1 R	1 R	2 R	2 R	1 R	1 R	1 R	2 R	2 R	2 R
Tobramycin	2 S	≤1 S	8 R	8 R	8 R	8 R	8 R	4 R	4 R	4 R
Trimethoprim/sulfamethoxazole	≤20 S	≤20 S	≤20 S	≤20 S	≤20 S	≤20 S	≤20 S	≤20 S	≤20 S	≤20 S

R – resistant; S – susceptible; I – intermediate

In contrast to this common pattern, minor strain-dependent differences were observed for selected antimicrobials. Resistance to piperacillin/tazobactam was detected in isolates 3 and 4, while the remaining strains were classified as susceptible. On the other hand, cefotaxime susceptibility was recorded only for isolates 1 and 2, whereas other isolates were resistant to this agent. A similar situation was observed for tobramycin. For cefepime and meropenem, most isolates exhibited intermediate susceptibility or susceptibility. Detailed antimicrobial susceptibility results, together with minimum inhibitory concentration values expressed in mg/L, are summarised in Table 3.

The results of sequence alignment analysis showed that all the gene fragments presented the highest similarity to the *S. marcescens* sequences deposited in the NCBI GenBank database. The query sequence coverage ranged between 95 and 100%, and the percentage of identical base pairs ranged from 88.3% for the 16S rRNA gene fragment to 99.71% for the *dinB1* sequence (Table 4). The results of molecular identification confirmed that the analysed bacterial isolate was *S. marcescens*.

**Table 4.** Basic local alignment search tool results for molecular identification of bacterial isolates from dairy-cow quarter-milk samples

Sequence	Query coverage	Percent identit	Accession length	Taxon	Accession No.
*rpoB*	95%	97.59%	5144137	*Serratia marcescens*	CP090244.1
*dinB1*	100%	99.71%	4962287	*Serratia marcescens*	CP132290.1
*dinB2*	96%	98.74%	4962287	*Serratia marcescens*	CP132290.1
16S rRNA	97%	88.30%	1220	*Serratia marcescens*	MH001477.1

## Discussion

The present study identified *Serratia marcescens* in pure cultures from mastitic quarters, which is consistent with a role in intramammary infection. The repeated isolation of this species from milk samples indicates its presence and potential role in mastitis cases under field conditions, although the available data do not allow the confirmation of a herd-level outbreak.

One of the general conclusions from this research is that the microbiological composition observed in the present study supports the complex and multifactorial aetiology of bovine intramammary infections. The dominance of streptococci, CNS and CPS is consistent with numerous reports identifying these groups as the primary causative agents of both clinical and subclinical mastitis in dairy herds worldwide (1, 10, 22). In particular, the high occurrence of CNS corresponds with the epidemiological shift observed in recent decades, whereby these organisms have emerged as prevalent mastitis pathogens following successful control programmes targeting *Staphylococcus aureus* (24).

Alongside the major mastitis pathogens, the isolation of *Enterococcus* spp. and other members of the Enterobacterales highlights the substantial contribution of environmental microorganisms to the epidemiology of intramammary infections. These bacteria are commonly associated with housing conditions, bedding materials and faecal contamination, and their presence has been linked to inadequate environmental hygiene and increased exposure of the teat end to environmental reservoirs (9, 13). Similarly, the sporadic detection of *Micrococcus* spp., *Bacillus* spp., *Aerococcus viridans* and *Candida* spp. aligns with previous studies describing these organisms as opportunistic or secondary pathogens, most often isolated in subclinical mastitis or mixed infections (3, 4, 23). Within this broader microbiological context, the detection of *Serratia* spp. deserves particular attention. Although *S. marcescens* is generally regarded as a minor mastitis pathogen, its involvement in intramammary infections is on a rising trend, particularly in association with environmental reservoirs such as water systems, bedding materials, disinfectant solutions and milking equipment (6, 11, 16). The recovery of *Serratia* isolates from different cows and during different sampling periods suggests a sporadic yet recurrent presence within the herd, rather than incidental contamination, a pattern that has previously been described in dairy farms affected by environmental mastitis outbreaks (2).

The occurrence of culture-negative samples and contaminated samples observed in this study is also consistent with routine mastitis diagnostics reported by other authors. Such findings are commonly attributed to intermittent bacterial shedding, low pathogen load, prior antimicrobial treatment or inherent limitations of culture-based diagnostic methods under field conditions (17, 18).

Building on these microbiological observations, the present study provided evidence for the involvement of *S. marcescens* as an aetiological agent of intramammary infections in several cows from the same herd. The pathogen was repeatedly isolated from milk samples and identified using a polyphasic diagnostic approach, including biochemical profiling with the VITEK 2 Compact system, MALDI-TOF mass spectrometry and species-specific PCR. The application of this comprehensive identification strategy is consistent with current recommendations for the diagnosis of uncommon or emerging mastitis pathogens and enhances the reliability of species-level identification (15, 26). The combined use of conventional biochemical methods, MALDI-TOF MS and molecular techniques provided convergent and robust evidence for the presence of *S. marcescens*. While MALDI-TOF MS is increasingly recognised as a rapid and accurate tool for routine identification of mastitis-associated bacteria (26), PCR-based assays offer an additional level of specificity, which is particularly important for differentiating *Serratia* spp. from closely related members of Enterobacterales (8). These findings support the integration of multiple diagnostic modalities when investigating atypical mastitis cases or suspected herd-level events.

Outbreaks of mastitis caused by *S. marcescens* have been described in dairy herds in several countries, and are most frequently associated with environmental contamination, including contamination of teat disinfectants, water sources and milking equipment (5, 20, 25). In agreement with these reports, the detection of *S. marcescens* in multiple cows in the same herd described here may suggest the presence of a common environmental reservoir or equipment-related source of infection. Although the specific source could not be conclusively identified in the present study, the temporal clustering of cases may support the interpretation of an epidemic pattern rather than isolated, unrelated infections. On the other hand, no molecular typing methods were applied in the presented study to assess the genetic relatedness of the isolates. As a result, it is not possible to determine whether the recovered strains represent a single clone or unrelated environmental strains. Consequently, the hypothesis of within-herd transmission or a common source cannot be confirmed and should be interpreted with caution. Given the limited number of isolates and the absence of epidemiological typing, the findings should be interpreted as evidence of occurrence rather than confirmation of an outbreak.

Considering the ubiquitous presence of *S. marcescens* in moist environments and its documented ability to form biofilms on milking equipment surfaces (13, 21), these findings underscore the importance of rigorous hygiene measures and routine microbiological surveillance of the milking system. This is particularly relevant because failure to prevent or detect colonisation at an early stage may result in infections and environmental contamination that are difficult to control, given the intrinsic resistance of *S. marcescens* to multiple β-lactam antibiotics and its reduced susceptibility to certain disinfectants (5). The *S. marcescens* isolates found in the presented research also shared a core profile of resistance to several β-lactams and other antimicrobial classes. Uniform resistance to early-generation cephalosporins and reduced susceptibility to colistin and tigecycline reflect the natural resistance background of S. marcescens, which limits therapeutic options in mastitis cases caused by this pathogen (5, 12, 25). From a practical standpoint, the conserved susceptibility to amikacin and ciprofloxacin suggests that these agents may retain activity against *S. marcescens* isolates associated with bovine mastitis; however, their use in food-producing animals must be carefully considered in the context of antimicrobial stewardship and regulatory restrictions (19). The observed resistance profile further supports the need to prioritise preventive measures and hygiene-based control strategies over antimicrobial therapy in managing environmental mastitis caused by opportunistic Gram-negative pathogens. Consequently, effective control of *S. marcescens* considered as a pathogen should proceed through preventive herd management strategies, including thorough cleaning and drying of milking clusters, regular monitoring of water quality and verification of disinfectant efficacy.

Finally, a limitation of the present investigation was the absence of environmental sampling, which prevented identification of the primary reservoir of *S. marcescens* on the farm. Future studies should incorporate systematic sampling of potential environmental sources, such as water systems, teat dips and milking equipment surfaces. This sampling should be coupled with molecular typing approaches to assess the genetic relatedness of isolates and to elucidate transmission pathways within affected herds.

## Conclusion

Despite all limitations, the study highlights the importance of including opportunistic environmental pathogens such as *S. marcescens* in routine mastitis diagnostics. The use of different identification approaches improved diagnostic accuracy and reduced the risk of misidentification. The findings also emphasise the need for appropriate hygiene management and targeted microbiological monitoring in dairy herds.
